# Relationships between Nut Size, Kernel Quality, Nutritional Composition and Levels of Outcrossing in Three Macadamia Cultivars

**DOI:** 10.3390/plants9020228

**Published:** 2020-02-11

**Authors:** Tarran E. Richards, Wiebke Kämper, Stephen J. Trueman, Helen M. Wallace, Steven M. Ogbourne, Peter R. Brooks, Joel Nichols, Shahla Hosseini Bai

**Affiliations:** 1 Genecology Research Centre, University of the Sunshine Coast, Maroochydore DC, QLD 4558, Australia; tarran.richards@corteva.com (T.E.R.); w.kaemper@griffith.edu.au (W.K.); helen.wallace@griffith.edu.au (H.M.W.); sogbourn@usc.edu.au (S.M.O.); pbrooks@usc.edu.au (P.R.B.); j.nichols2@griffith.edu.au (J.N.); shossein@usc.edu.au (S.H.B.); 2 Department of Animal Ecology, Evolution and Biodiversity, Ruhr-University Bochum, 44780 Bochum, Germany; 3 Environmental Futures Research Institute, School of Environment and Science, Griffith University, Nathan, Brisbane, QLD 4111, Australia; 4 School of Health, Medical and Applied Sciences, CQ University, Bundaberg, QLD 4670, Australia

**Keywords:** breeding system, fatty acids, health, kernels, macadamia, mating system, nutrients, nuts, pollination, self-incompatibility

## Abstract

Tree nuts play an important role in healthy diets, but their economic value and nutritional quality may be affected by their size and paternity. We assessed relationships between nut size and kernel recovery, the incidence of whole kernels, fatty acid composition and mineral nutrient concentrations in three macadamia cultivars, “Daddow”, “816” and “A4”. We determined to what extent differences in nut size and quality were the result of different levels of cross- or self-paternity. Small nuts of all cultivars had lower kernel recovery than large nuts, and small nuts provided lower incidence of whole kernels in “Daddow” and “A4”. Small kernels had a lower relative abundance of the saturated fatty acid, palmitic acid, in all cultivars and higher relative abundance of the unsaturated fatty acid, oleic acid, in “Daddow” and “A4”. Small kernels had higher concentrations of many essential nutrients such as nitrogen and calcium, although potassium concentrations were lower in small kernels. Most nuts arose from cross-pollination. Therefore, nut size and kernel quality were not related to different levels of cross- and self-paternity. Identified cross-paternity was 88%, 78% and 90%, and identified self-paternity was 3%, 2% and 0%, for “Daddow”, “816” and “A4”, respectively. Small macadamia kernels are at least as nutritious as large macadamia kernels. High levels of cross-paternity confirmed that many macadamia cultivars are predominantly outcrossing. Macadamia growers may need to closely inter-plant cultivars and manage beehives to maximise cross-pollination.

## 1. Introduction

Tree nuts play an important role in healthy human diets, providing beneficial fatty acids, proteins and essential mineral nutrients [1,2,3]. Most tree nuts are high in unsaturated fatty acids and low in saturated fatty acids [2,3,4,5,6,7,8,9]. Foods rich in unsaturated fatty acids reduce serum cholesterol and low-density lipoprotein (LDL) cholesterol levels and reduce the risk of cardiovascular disease [2,10,11,12,13,14]. Tree nuts provide high levels of amino acids and non-sodium mineral nutrients, including L-arginine and magnesium, that decrease inflammation, blood pressure and oxidative stress [2,7,15]. Tree nuts are also a valuable source of calcium, iron and zinc, which can be absorbed insufficiently during phases of high dietary demand such as infancy, childhood, adolescence, pregnancy and breastfeeding [16,17,18,19,20,21].

The fatty acid composition and nutrient concentrations of tree-nut kernels can vary greatly among cultivars [9,22,23,24,25,26,27,28]. Fatty acid composition and nutrient concentrations can also vary within almond or pistachio cultivars because of differences in the pollen parentage of individual kernels [22,23,24,25,29,30]. For example, self-pollinated kernels of some almond cultivars have lower oleic:linoleic acid ratios than cross-pollinated kernels [25,29]. In addition, self-pollinated kernels of some almond, chestnut and hazelnut cultivars are smaller than cross-pollinated kernels [22,31,32,33]. Such effects of the paternal genetic background on the size or quality of the embryo are termed “xenia”, and xenic effects are common in fruit and nut crops [34]. Therefore, maximising both yield and human-health benefits may depend upon ensuring high levels of cross-pollination in tree-nut orchards.

Relationships between kernel size, nutritional composition, and cross- or self-paternity are poorly understood for some nuts, including the subtropical tree nut, macadamia (*Macadamia integrifolia*, *M. tetraphylla* and hybrids). Macadamia flowers are bee-pollinated and partially self-incompatible [35,36,37,38,39,40,41,42,43]. Manual cross-pollination of macadamia racemes produces more fruit than manual self-pollination [44,45]. Nut-in-shell (NIS) mass, kernel mass, and the ratio of kernel:shell (i.e., kernel recovery) also vary between macadamia fruit arising from open-pollination and supplementary cross-pollination [45,46]. The differences in nut quality (of cv. “660”) were attributed to differences between self- and cross-pollination [46]. However, subsequent paternity analyses revealed that 80%–100% of nuts in a 27-row single-cultivar block of “A16” trees were cross-pollinated [47] and that 60%–100% of nuts from nine cultivars in a multi-cultivar trial were also cross-pollinated [48]. Therefore, variations in nut and kernel size among pollination treatments in early studies might not have been the result of different levels of self- and cross-pollination. Thus, we currently know very little about how self- and cross-pollination of macadamia flowers affect basic kernel-quality attributes such as kernel mass, kernel recovery, and the incidence of whole kernels. We also know very little about how pollen parentage affects the fatty acid composition and nutrient concentrations of macadamia kernels.

In this study, we selectively sampled small and large nuts from three macadamia cultivars, hypothesising that small nuts had arisen from self-pollination, whereas large nuts had arisen from cross-pollination. We determined how nut size was related to kernel recovery, the incidence of whole kernels, fatty acid composition and mineral nutrient concentrations. We then assessed the extent to which differences in nut size and kernel quality were the result of different levels of cross- and self-paternity.

## 2. Results and Discussion

### 2.1. Kernel Recovery and Incidence of Whole Kernels

Small nuts of all three cultivars had lower kernel mass and kernel recovery than large nuts (Table 1). These relationships between nut and kernel parameters are consistent with previous results within cultivars “A4”, “246” and “660” [45,46]. Strong positive correlations have also been found between nut mass and kernel mass across macadamia cultivars, although non-significant or negative correlations have been detected between nut mass and kernel recovery [49,50]. The average kernel recovery from Australian macadamia orchards is approximately 34% and growers receive a premium for producing nuts with higher kernel recovery [51,52]. Our results demonstrate that maximising nut size within cultivars can increase kernel yield and kernel value, which could improve financial returns to growers.

Supplementary cross-pollination, compared with open-pollination, has increased kernel recovery previously by 1.5%–3.3% in cultivars “660”, “246” and “A4” [45,46]. These increases are similar to the differences we detected between small and large nuts (Table 1). The final sizes of the macadamia kernel and nut-in-shell are determined halfway through fruit development when the shell hardens after the rapid growth of the endosperm and embryo [53,54,55,56,57,58]. The endosperm and embryo, which comprise the immature kernel, are both progeny tissues (i.e., combining maternal and paternal alleles) and so it was possible that kernel size was affected directly by fruit paternity [58,59].

Small “Daddow” and “A4” nuts also yielded a lower percentage of whole kernels than did large nuts (Table 1). The incidence of whole kernels did not differ significantly between small and large nuts of cultivar “816” (Table 1). Correlations between whole-kernel incidence and either nut mass, kernel mass or kernel recovery across 40 cultivars have previously been found non-significant [49]. In addition, whole-kernel incidence had not been related previously to nut size within individual cultivars. However, some cultivars such as “A16”, “A38” and “816” that tend to have large kernels and high kernel recovery [60,61,62,63,64] do provide a high percentage of whole kernels [65,66,67]. Macadamia processors trade kernels under a range of “styles”, with the highest-value styles (0, 1 and 2) comprised predominantly of whole kernels rather than halves or pieces [67,68]. Our results further demonstrate that maximising nut size can increase kernel value, which could improve financial returns to macadamia processors.

### 2.2. Fatty Acid Composition

Kernels from small nuts of all three cultivars had a lower relative abundance of the saturated fatty acid (SFA), palmitic acid, than kernels from large nuts (Table 2). These reductions were small (0.38% to 0.76% relative abundance) and they were offset in “816” and “A4” kernels by increases in the relative abundance of another SFA, stearic acid. Kernels of small “Daddow” and “A4” nuts had higher relative abundance of the predominant unsaturated fatty acid (UFA), oleic acid (Table 2). This was offset partly in “Daddow” by lower relative abundance of another UFA, palmitoleic acid (Table 2), of which macadamia is one of the main possible dietary sources [69]. Differences in the relative abundances of other fatty acids were negligible (Table 2). Small “Daddow” kernels had lower total-SFA relative abundance, higher total-UFA relative abundance and, thus, a higher UFA:SFA ratio than large kernels (Table 3). The differences in total-SFA and total-UFA relative abundance were less than 1% but they could confer a slight health advantage on small “Daddow” kernels because of the beneficial effects of dietary UFAs in regulating lipid levels, maintaining healthy body weight and preventing inflammation [12,13,18,70]. Total-SFA relative abundance, total-UFA relative abundance and UFA:SFA ratio did not differ significantly between small and large kernels of “816” or “A4” (Table 3). However, partial replacement of one SFA, palmitic acid, with another SFA, stearic acid, in the small kernels of “816” and “A4” (Table 2) might provide a slight health advantage because palmitic acid increases LDL-cholesterol levels whereas stearic acid does not [13,71].

Our results showed that small macadamia kernels have a fatty acid profile that is at least as healthy as large macadamia kernels. However, the differences in fatty acid profile between small and large kernels were much less than the differences between macadamia cultivars [27,72,73] and between tree–nut species including almond, hazelnut, macadamia, pecan, pistachio and walnut [4,9]. All these nuts are considered beneficial for reducing the risk of cardiovascular disease because of their high levels of unsaturated fatty acids [1,2,7,10,14,74,75,76,77,78,79].

### 2.3. Mineral Nutrient Concentrations

Kernels from small nuts had higher concentrations of many mineral nutrients than those from large nuts (Table 4). Nutrient concentrations were similar to standard levels for unroasted macadamia kernels [80] although copper concentrations, not surprisingly, were low for kernels produced on the coastal dermosol and kandosol soils of Sites 1 and 2 and manganese levels were low, especially for kernels produced on the kandosol soil at Site 2 [81]. The total contents of each of the 13 nutrients were always lower in kernels from small nuts than large nuts (data not presented). Nitrogen concentrations were 6%–8% higher in kernels from small nuts (Table 4). Nitrogen concentrations are correlated with protein concentrations in plant-foodstuffs [82], and nuts are often consumed as an important source of protein [21,83,84,85]. Small kernels had 20%–29% higher calcium concentrations than large kernels (Table 4). Small kernels also had 22% and 14% higher iron concentrations than large kernels in “Daddow” and “A4”, respectively, and 11% higher zinc concentration than large kernels in “816” (Table 4). Nuts are recommended as a source of calcium, iron and zinc, which are often absorbed in insufficient levels, particularly during infancy, childhood, adolescence, pregnancy and breastfeeding [16,17,19,20,21,85,86,87,88,89]. Magnesium concentrations were 9% higher in kernels from small nuts than large nuts of “Daddow” and “816” (Table 4). Nuts are also considered a good source of magnesium, and higher serum magnesium levels are associated with lower blood pressure, inflammation, vascular calcification and oxidative stress, and reduced risk of cardiovascular disease [2,7,90,91,92]. Importantly, sodium concentrations did not differ significantly between small and large macadamia kernels (Table 4). Therefore, dietary salt intake would not be affected by the selection of unsalted macadamia based on kernel size, assuming equivalent-mass consumption.

Potassium concentrations, in contrast to the other mineral nutrients, were lower (by 8%–12%) in kernels from small nuts (Table 4). These results might relate to the phloem mobility of potassium. Fruit calcium concentrations are often strongly negatively related to fruit mass whereas potassium concentrations are often more-weakly negatively related, or not related significantly, to fruit mass [93,94,95,96,97]. However, positive relationships between fruit potassium concentration and fruit mass have sometimes been detected in apple and avocado [94,95]. These relationships were largely between mineral nutrient concentrations and mass of maternal flesh tissue (i.e., non-embryonic tissue) whereas the current relationships in macadamia kernels relate entirely to embryonic tissue.

### 2.4. Levels of Cross- and Self-Paternity

Both small and large macadamia nuts arose mostly from cross-pollination (Table 5). The overall levels of cross-paternity were at least 88% ± 2%, 78% ± 2% and 90% ± 1% for “Daddow”, “816” and “A4”, respectively. Identified levels of self-paternity were 3% ± 1%, 2% ± 1% and 0% for the same respective cultivars. Therefore, differences in kernel recovery, whole-kernel incidence, fatty acid composition and mineral nutrient concentrations were not due to differences in the levels of cross- or self-paternity, as both small and large nuts were mostly cross-pollinated. The high levels of cross-paternity in mature nuts were surprising because macadamia flowers are only partially self-incompatible [37,44,45,46,98]. Self-pollen tubes are often arrested in the upper style of macadamia flowers whereas cross-pollen tubes have a higher likelihood of penetrating to the lower style and initiating fruit set [35,37,99]. Most initially-set fruit abscise during the first half of the fruit development period [46,55,57,100] and so it is possible that self-pollinated fruit are shed selectively during this period of immature fruit drop [101,102,103].

The high level of cross-paternity in “Daddow” was particularly interesting because “Daddow” nuts were sampled in the middle of a 48-row single-cultivar block. Here, at least 88% of nuts were fathered by trees that were at least 200 m away. These results extend previous findings that 85%–90% of nuts in the middle of a 27-row block of “A16” trees arose from cross-pollination [47]. In that case, most nuts were fathered by trees that were at least 98 m away. More recently, 60%–100% of nuts from nine different cultivars in a closely-interplanted multi-cultivar trial were found to be cross-pollinated, with nuts of five of these cultivars, “A16”, A4”, “246”, “344” and “800”, displaying 100% cross-paternity [48]. These results, in combination, demonstrate that the realised mating system of many macadamia cultivars is predominantly outcrossing. It remains possible that some cultivars may have lower levels of outcrossing, in which case further research could determine whether nuts arising from cross- and self-pollination differ in their nut size, kernel quality and nutritional composition.

The finding that many macadamia cultivars are predominantly outcrossing has enormous implications for the management and design of macadamia orchards. Few macadamia growers rank pollination as a major factor that limits production, with most Australian growers nominating more-clearly observable factors such as storm and hail damage, pest outbreaks, and hot or dry weather [52]. Macadamia cultivars are rarely inter-planted within the same rows in orchards. Instead, orchards are typically established with a system of single-cultivar blocks [40,47] that could be, for example, 5 rows wide (as at Site 2) or 48 rows wide (as at Site 1). However, macadamia flowers are bee-pollinated [36,38,40,41,42,43,45,98] and we have demonstrated that nut production is almost totally reliant on the transfer of pollen from one cultivar to another. Therefore, there is the potential for yield to decline with increasing distance from bee hives [104,105,106] or with increasing distance from another cultivar, as found previously in a 27-row block of “A16” trees [40,47].

## 3. Materials and Methods

### 3.1. Study Sites

We sampled nuts from two commercial macadamia orchards near Bundaberg, Queensland, Australia. Bundaberg has a humid subtropical climate with wet summers and dry winters, mean annual rainfall of 1009.5 mm, and mean minimum–maximum temperatures of 21.5–30.4 °C in January and 10.3–22.3 °C in July [107]. Site 1 (24^°^47′53″ S 152^°^17′36″ E) had a red-dermosol soil, while Site 2 (24^°^56′6″ S 152^°^21′16″ E) had either a yellow/brown kandosol or a red kandosol soil depending on the location within the orchard [81]. Trees at both sites received fertigation via under-tree sprinklers that were suspended from irrigation pipes. Site 1 contained cultivars “Daddow” and “816”, approximately 10 years old, each planted in pure blocks of a single cultivar that were 48 and 42 rows wide, respectively. Rows were 8 m apart and trees within each row were 4 m apart. Site 2 contained cultivars, “A4”, “A16”, “A29”, “A38”, “A203”, “A268”, “Daddow”, “Own Venture”, “246”, “344”, “660”, “741”, “814”, “816”, “835”, “842” and “849”, approximately 13 or 16 years old, depending on location within the orchard. Trees were planted in blocks containing multiple cultivars, with each cultivar occupying five or ten contiguous rows, depending on whether each second row was yet to be removed as the trees aged. Tree spacing was 5 or 10 m between rows and 2 m within a row. Sampled trees were in an area where the between-row spacing was now 10 m.

### 3.2. Sampling Design, Sample Collection and Processing

We investigated three cultivars, with each cultivar sampled at one site. Cultivar “Daddow” was sampled at Site 1. Cultivars “816” and “A4” were sampled at Site 2. “Daddow” trees are typically large and rounded, with an open canopy that progresses to a dense canopy with age, and with medium-sized nuts [60]. “816” trees are large and upright with a dense canopy and medium to large nuts [60]. “A4” trees are medium-sized with an open spreading habit, and with large to very-large nuts [60].

The middle row of each cultivar was selected to increase the likelihood of finding self-pollinated nuts. We sampled six trees from each cultivar, commencing with the fifth tree from the end of each row at Site 1 and the twentieth tree from the end of each row at Site 2, and then sampling every tenth tree. We collected samples from the orchard floor under each tree on two occasions during the harvest season of each orchard. “Daddow” nuts were collected at Site 1 on 26 Mar 2018 and 18 Apr 2018. Cultivar “816” and “A4” nuts were collected at Site 2 on 27 Mar 2018 and 10 May 2018.

We dehusked the fruit from each sampled tree before drying the nuts at 37 °C for 2 d, 45 °C for 2 d and 57 °C for 2 d [108]. Non-commercial nuts that were too small to be cracked using commercial crackers (<18 mm in diameter) were excluded. The remaining nuts from each harvest were sorted into two size categories, selecting the ten smallest nuts and the ten largest nuts based on visual examination of nut diameter. This resulted in a total of 240 nuts (6 trees × 2 harvests × 2 sizes × 10 nuts) from each cultivar.

We recorded nut-in-shell (NIS) mass for each nut, before cracking it using a manual nutcracker (T.J’s, Morayfield, Australia). Kernel mass, and whether the kernel remained whole or split into halves, was also recorded. Kernel recovery was calculated as the percentage of NIS that was comprised of kernel. Each individual kernel was then crushed and dissected into three representative sub-samples of at least: (a) 500 mg for fatty acid analysis; (b) 300 mg for mineral nutrient analysis; and (c) 30 mg for paternity analysis.

### 3.3. Fatty Acid Analysis

We analysed fatty acids as their methyl esters from 154 “Daddow”, 146 “816”, and 160 “A4” kernels. The first 144 kernels of each cultivar were selected randomly, and additional kernels were selected based on known paternity (see Section 3.5, below). The sub-sample of crushed dried kernel was crushed further to extract oil. Crushed sub-samples were added to 25 mL of pentane, magnetically stirred for 15 min, and then centrifuged for 5 min at 2500 rpm to remove suspended solids from the pentane. Pentane was evaporated from the oil using an air-tight vacuum rotator for 5–10 min. The extracted oil was stored in glass vials at 4 °C [6].

We added 0.7 mL of anhydrous methanol dibutyl hydroxytoluene solution (0.015%, *v/v*) and 25 µL of HCl (32%, *v/v*) to 1 µL of the extracted oil. The mixture was incubated for at least 20 h at 65 °C before adding 0.5 mL of hexane and 0.5 mL of deionised water and shaking for 30 sec. The layer of deionised water was removed manually before adding another 0.5 mL of deionised water to the hexane. The layer of deionised water was removed, and anhydrous Na_2_SO_4_ was added to remove any remaining water from the hexane. The hexane was removed, avoiding disruption of the Na_2_SO_4_ residue, and fatty acids were analysed by gas chromatography–mass spectrometry (GC–MS; PerkinElmer Clarus 580 GC coupled to a PerkinElmer Clarus SQ8S MS) (Waltham, MA, USA) [3]. The GC column was an Elite-5MS (30 m × 0.25 mm × 0.25 µm). The helium carrier gas had a constant flow of 1 mL/min. The injection port was at 260 °C with a split ratio of 50:1. The temperature program was 50 °C for 0.5 min, ramping at 10 °C/min until 300 °C, and holding for 1.0 min. The MS analysed a mass range from 40 to 400 (m/z), from 3.1 to 26.5 min at 70 eV. We identified compounds by comparing retention times with authentic standards and comparing mass spectra against National Institute of Standards and Technology (NIST) (08) MS reference-library matches. Quantitation of each compound was via integration of the peak area at the authentic-standard retention time on the total ion current chromatogram.

### 3.4. Mineral Nutrient Analysis

We determined the concentration of nitrogen (N) by combustion analysis using a LECO CNS 2000 (LECO, Saint Joseph, MI, USA) [109,110]. We determined aluminium (Al), boron (B), calcium (Ca), copper (Cu), iron (Fe), magnesium (Mg), manganese (Mn), phosphorus (P), potassium (K), sodium (Na), sulphur (S) and zinc (Zn) concentrations by inductively coupled plasma–atomic emission spectroscopy after nitric and perchloric acid digestion [111,112].

### 3.5. Paternity Analysis

We collected a leaf from each sampled tree, from which an approximately 50 mg subsample was used to confirm cultivar identity. We also identified potential pollen–parent cultivars within 1.5 km of the study sites, and we collected leaf samples from each of these cultivars. Seventeen cultivars were identified as candidate pollen parents and incorporated into a reference library for paternity assignment. This reference library contained no additional cultivars to those established in the orchard at Site 2; i.e., candidate pollen–parent cultivars were “A4”, “A16”, “A29”, “A38”, “A203”, “A268”, “Daddow”, “Own Venture”, “246”, “344”, “660”, “741”, “814”, “816”, “835”, “842” and “849”.

We froze each leaf or kernel subsample in liquid nitrogen and ground it to a powder using an MM2000 TissueLyser (Retsch, Haan, Germany) after adding disposable 0.1 and 2.3 mm diameter zirconia/silica beads (Daintree Scientific, St. Helens, Tasmania, Australia) [113]. DNA was extracted using a glass-fibre DNA-extraction protocol for plants [114]. DNA of each subsample was amplified at four different microsatellite loci [115] (Table 6) in a Mastercycler (Eppendorf, Hamburg, Germany). The 5′ end of one primer of each primer pair was fluorescently labelled. Multiplex PCR was carried out for microsatellite loci Mac001, Mac002 and Mac005 using a Type-it microsatellite PCR kit (Qiagen, Hilden, Germany). Microsatellite loci were amplified in 12.5 μL reaction volumes containing approximately 20 ng DNA template, 0.2 μM of each oligonucleotide primer, 1 × Type-it Multiplex PCR Master Mix and 3 μL sterile water. Multiplex PCR was performed with initial denaturation at 95 °C for 5 min, followed by 35 cycles of 95 °C for 30 s, 57 °C for 1 min and 72 °C for 1 min, then final elongation at 60 °C for 30 min. Samples were maintained at 10 °C. Microsatellite locus Mac006 was amplified in 11.5 μL reaction volumes containing approximately 20 ng DNA template, 1.2 × F1 Taq reaction buffer, 0.02 U F1 Taq (Fisher Biotec, Wembley, Australia), 0.1 mM dNTPs, 2.4 mM MgCl_2_, 0.2 μM of each oligonucleotide primer and 6.7 μL sterile water. PCR was performed with initial denaturation at 94 °C for 2 min, followed by 35 cycles of 94 °C for 10 s, 55 °C for 10 s and 70 °C for 1 min, then final elongation at 70 °C for 5 min. Samples were maintained at 10 °C.

We separated the PCR products by agarose gel electrophoresis and viewed them under ultra-violet light on a Gelscan visualiser (Sebia, Lisse, France). PCR products were diluted with molecular-grade water (1:19, v:v). The PCR dilution (1 μL) was added to a solution containing 9 μL Hi-Di and 0.1 μL GS-600 LIZ (Applied Biosystems, Foster City, CA, USA) and injected into an AB 3500 Genetic Analyser (Thermo Fisher Scientific, Waltham, MA, USA). We determined allele sizes relative to GS-600 LIZ using GeneMarker v.2.6.3 (SoftGenetics, State College, PA, USA). Scoring was cross-checked manually to ensure accuracy and consistency [116]. We identified two different genotypes in cultivar “A203” and “741” leaf samples and so these were labelled as either “A203_1”, “A203_2”, “741_1” or “741_2” in the reference library. This provided 19 potential pollen parents for each nut.

We analysed paternity of kernels by the logarithm of odds (LOD) method using Cervus 2.0 software (Field Genetics Ltd., London, UK). The LOD for each possible pollen parent was calculated by taking the natural log of the overall likelihood ratio. A positive LOD meant that the candidate cultivar was more likely to be the true pollen parent than a cultivar selected randomly from the reference library. Cultivars with a negative LOD were excluded as pollen parents for each kernel. We then assigned nuts as cross- or self-pollinated if all remaining pollen parents were either cross-pollen cultivars or the self-pollen cultivar, respectively. Remaining nuts were then assigned as cross- or self-pollinated if both cross- and self-cultivars were possible pollen parents, but one candidate pollen parent had strict-level confidence (95%) of the LOD. Kernels were considered “unassigned” if both cross- and self-pollination remained possible but the confidence value for the primary-candidate pollen parent was less than 95%. Kernels were considered “mismatched” if no parent pair from the reference library could explain its genotype.

### 3.6. Statistical Analysis

We analysed data for nut-in-shell mass, kernel mass, kernel recovery, fatty-acid relative abundance, kernel nutrient concentrations and kernel nutrient contents by 3-way analyses of variance (nut size × harvest time × tree) using IBM SPSS Statistics v. 25. Nut size × harvest time and nut size × harvest time × tree interactions were generally not significant and so reported data were pooled across the two harvest times. Whole kernel and paternity data were analysed by paired *t*-tests. The data sets had normal distributions and homogeneous variances, and so data transformation was unnecessary. Means and interactions were regarded as significantly different at *p* < 0.05. Means are reported with standard errors.

## 4. Conclusions

Small macadamia nuts provided lower kernel recovery and whole-kernel incidence than did large nuts, which reduces their financial value. However, higher mineral nutrient concentrations and more nutritionally-beneficial fatty acid profiles ensured that kernels from small macadamia nuts were at least as healthy in human diets as kernels from large nuts. Relationships between nut size and kernel quality were not the result of differences in cross- and self-paternity levels between small and large nuts. Macadamia trees were predominantly outcrossing. Most nuts of all three cultivars, “Daddow”, “816” and “A4”, were cross-pollinated, even in the middle of a 48-row block of a single cultivar. Growers might need to consider inter-planting cultivars closely and managing bee hives to maximise the dispersal of pollen from one macadamia cultivar to another.

## Figures and Tables

**Table 1:** Relationships between nut size, kernel size, kernel recovery, and incidence of whole kernels in macadamia cultivars, “Daddow”, “816” and “A4”, each sampled at one of two commercial orchard sites.

	Cultivar and Nut Size					
	“Daddow” (Site 1)		“816” (Site 2)		“A4” (Site 2)	
	Small	Large	Small	Large	Small	Large
Nut-in-Shell Mass (g)	4.75 ± 0.05a	8.59 ± 0.10b	5.17 ± 0.08a	8.79 ± 0.08b	6.33 ± 0.09a	9.97 ± 0.27b
Kernel Mass (g)	1.72 ± 0.03a	3.31 ± 0.03b	2.20 ± 0.05a	4.06 ± 0.04b	2.65 ± 0.06a	4.29 ± 0.07b
Kernel Recovery (%)	35.9 ± 0.4a	38.8 ± 0.3b	42.5 ± 0.7a	46.2 ± 0.4b	41.6 ± 0.8a	43.7 ± 0.6b
Whole Kernels (%)	60.0 ± 2.4a	76.0 ± 3.5b	79.0 ± 3.6	82.0 ± 2.4	64.0 ± 2.7a	72.0 ± 2.4b

Means ± SE with different letters within a cultivar are significantly different (3-way ANOVA for nut-in-shell mass, kernel mass and kernel recovery, and paired *t*-test for whole kernels; *p* < 0.05, *n* = 237–240 nuts except *n* = 6 trees for whole kernels).

**Table 2:** Relationships between nut size and relative abundances (%) of fatty acids in kernels from macadamia cultivars, “Daddow”, “816” and “A4”, each sampled at one of two commercial orchard sites.

Fatty Acid	Cultivar and Nut Size					
	“Daddow” (Site 1)		“816” (Site 2)		“A4” (Site 3)	
	Small	Large	Small	Large	Small	Large
Myristic Acid (C14:0)	0.51 ± 0.02	0.51 ± 0.02	0.60 ± 0.02	0.61 ± 0.02	0.29 ± 0.01	0.28 ± 0.01
Palmitoleic Acid (C16:1 cis)	17.57 ± 0.44a	19.55 ± 0.37b	17.18 ± 0.34	17.38 ± 0.34	18.38 ± 0.30	19.10 ± 0.29
Palmitic Acid (C16:0)	9.30 ± 0.12a	10.06 ± 0.11b	9.75 ± 0.15a	10.26 ± 0.14b	9.39 ± 0.11a	9.77 ± 0.10b
Linoleic Acid (C18:2)	1.04 ± 0.05	1.13 ± 0.06	1.02 ± 0.05	0.95 ± 0.04	0.91 ± 0.04a	1.02 ± 0.03b
Oleic Acid (C18:1 cis)	59.93 ± 0.53a	57.14 ± 0.42b	60.10 ± 0.39	60.12 ± 0.44	57.83 ± 0.37a	56.98 ± 0.36b
Elaidic Acid (C18:1 trans)	4.72 ± 0.10	4.55 ± 0.09	3.74 ± 0.07	3.58 ± 0.06	4.52 ± 0.09	4.64 ± 0.08
Stearic Acid (C18:0)	3.24 ± 0.08	3.28 ± 0.10	3.79 ± 0.11a	3.43 ± 0.09b	4.76 ± 0.14a	4.34 ± 0.14b
Eicosenoic Acid (C20:1)	1.70 ± 0.04	1.70 ± 0.04	1.56 ± 0.03	1.54 ± 0.03	1.27 ± 0.04	1.34 ± 0.04
Arachidic Acid (C20:0)	1.99 ± 0.04	2.07 ± 0.04	2.27 ± 0.05a	2.14 ± 0.03b	2.66 ± 0.05	2.52 ± 0.06

Means ± SE with different letters within a cultivar are significantly different (3-way ANOVA, *p* < 0.05, *n* = 146–160 nuts).

**Table 3:** Relationships between nut size, relative abundances of fatty acids (%), and fatty acid ratios in kernels from macadamia cultivars, “Daddow”, “816” and “A4”, each sampled at one of two commercial orchard sites.

Fatty Acids	Cultivar and Nut Size					
	“Daddow” (Site 1)		“816” (Site 2)		“A4” (Site 3)	
	Small	Large	Small	Large	Small	Large
Saturated (%)	15.05 ± 0.16a	15.93 ± 0.14b	16.41 ± 0.20	16.43 ± 0.20	17.06 ± 0.18	16.92 ± 0.21
Unsaturated (%)	84.95 ± 0.16a	84.07 ± 0.14b	83.60 ± 0.20	83.57 ± 0.20	82.94 ± 0.18	83.08 ± 0.21
Unsaturated:Saturated	5.70 ± 0.07a	5.31 ± 0.05b	5.18 ± 0.10	5.15 ± 0.08	4.91 ± 0.06	4.98 ± 0.07

Means ± SE with different letters within a cultivar are significantly different (3-way ANOVA, *p* < 0.05, *n* = 146–160 nuts).

**Table 4:** Relationships between nut size and mineral nutrient concentrations (mg/100g) in kernels from macadamia cultivars, “Daddow”, “816” and “A4”, each sampled at one of two commercial orchard sites.

Nutrient^1^	Cultivar and Nut Size					
	“Daddow” (Site 1)		“816” (Site 2)		“A4” (Site 2)	
	Small	Large	Small	Large	Small	Large
N	1530 ± 10a	1450 ± 10b	1600 ± 10a	1500 ± 10b	1540 ± 10a	1430 ± 10b
Al	0.38 ± 0.03	0.39 ± 0.04	0.32 ± 0.02	0.30 ± 0.01	0.43 ± 0.03a	0.31 ± 0.01b
B	1.08 ± 0.06a	0.93 ± 0.03b	0.77 ± 0.04	0.77 ± 0.04	1.10 ± 0.06a	0.88 ± 0.04b
Ca	53.07 ± 1.73a	44.39 ± 1.38b	59.03 ± 1.74a	48.54 ± 1.24b	74.59 ± 2.06a	57.92 ± 1.22b
Cu	0.28 ± 0.01a	0.25 ± 0.01b	0.37 ± 0.01a	0.33 ± 0.01b	0.35 ± 0.01	0.33 ± 0.01
Fe	3.04 ± 0.09a	2.49 ± 0.07b	2.30 ± 0.09	2.08 ± 0.08	2.26 ± 0.12a	1.97 ± 0.04b
K	404.99 ± 8.97a	439.90 ± 8.40b	368.80 ± 6.26a	403.71 ± 6.69b	370.95 ± 8.65a	423.04 ± 7.04b
Mg	145.05 ± 2.12a	130.77 ± 1.88b	128.82 ± 1.96a	117.81 ± 1.53b	119.27 ± 1.95	115.50 ± 1.63
Mn	2.58 ± 0.11a	1.93 ± 0.08b	0.52 ± 0.03	0.48 ± 0.02	0.64 ± 0.03a	0.56 ± 0.03b
Na	6.11 ± 0.41	5.90 ± 0.39	7.53 ± 0.42	8.02 ± 0.41	8.87 ± 0.97	8.75 ± 0.94
P	261.84 ± 3.27a	245.23 ± 2.94b	234.35 ± 3.05a	205.03 ± 3.05b	234.68 ± 3.80a	221.86 ± 3.26b
S	142.49 ± 2.06a	131.78 ± 1.83b	132.61 ± 2.24a	113.53 ± 2.22b	157.16 ± 2.49a	150.69 ± 1.88b
Zn	1.79 ± 0.04	1.81 ± 0.05	1.44 ± 0.05a	1.29 ± 0.04b	1.68 ± 0.06	1.64 ± 0.05

^1^ Nitrogen (N), aluminium (Al), boron (B), calcium (Ca) copper (Cu), iron (Fe), potassium (K), magnesium (Mg), manganese (Mn), sodium (Na), phosphorous (P), sulphur (S), zinc (Zn). Means ± SE with different letters within a cultivar are significantly different (3-way ANOVA, *p* < 0.05, *n* = 237–240 nuts).

**Table 5:** Relationships between nut size and identified levels of cross- and self-paternity in macadamia cultivars, “Daddow”, “816” and “A4”, each sampled at one of two commercial orchard sites.

Paternity	Cultivar and Nut Size					
	“Daddow” (Site 1)		“816” (Site 2)		“A4” (Site 2)	
	Small	Large	Small	Large	Small	Large
Cross (%)	93 ± 2	82 ± 3	80 ± 2	75 ± 3	89 ± 2	91 ± 2
Self (%)	5 ± 2	2 ± 1	4 ± 2	0	0	0

Means ± SE between small and large nuts within a cultivar do not differ significantly (paired *t*-test, *p* > 0.05, *n* = 6 trees). Remaining nuts could not be assigned as either cross- or self-pollinated with a strict (95%) level of confidence.

**Table 6:** Polymorphic microsatellite loci used to determine paternity of macadamia kernels^1^.

Locus		Primer Sequences (5′–3′)	Repeat Motif	Fluorescent Label	Allele Size Range (bp)
Mac001	F:	GTGACTGGTGGACACCAAAACCCA	(AT)_11_	VIC	407–429
	R:	GCACTAGGTGTCACCCCCACTTCT			
Mac002	F:	CCCAACTGGGTTTGCAAGGACCAA	(CT)_8_	NED	271–313
	R:	AGTAGCCGCGAGCTGATCGAAGAT			
Mac005	F:	CATAGCATGAGTTTCAAGGGATAA	(AAG)_10_	FAM	255–356
	R:	ATTACAAACCCACTCTTCGATTT			
Mac006	F:	TTTCATCATTGATCATCATAGGTACA	(AG)_11_	PET	314–368
	R:	GAGCTAATACTTAACCAGGTGAACA			

^1^ Loci identified and primers developed previously for macadamia [115].
